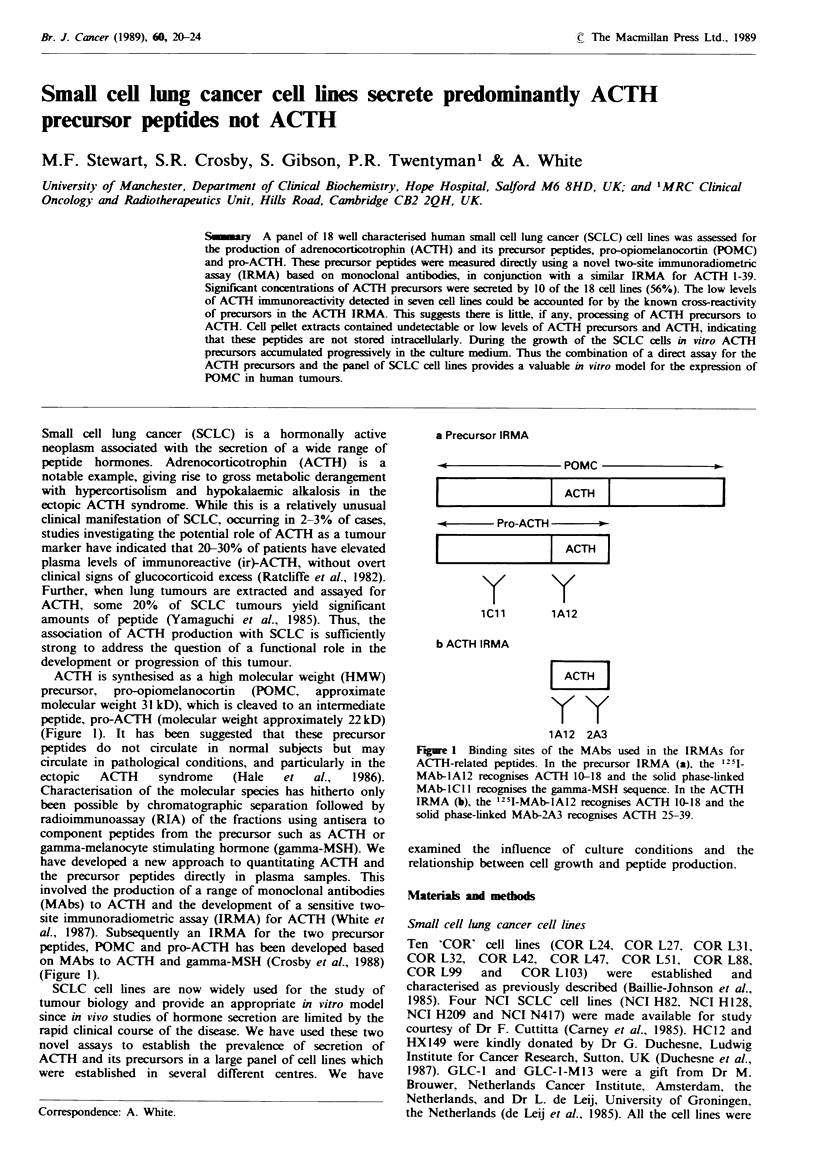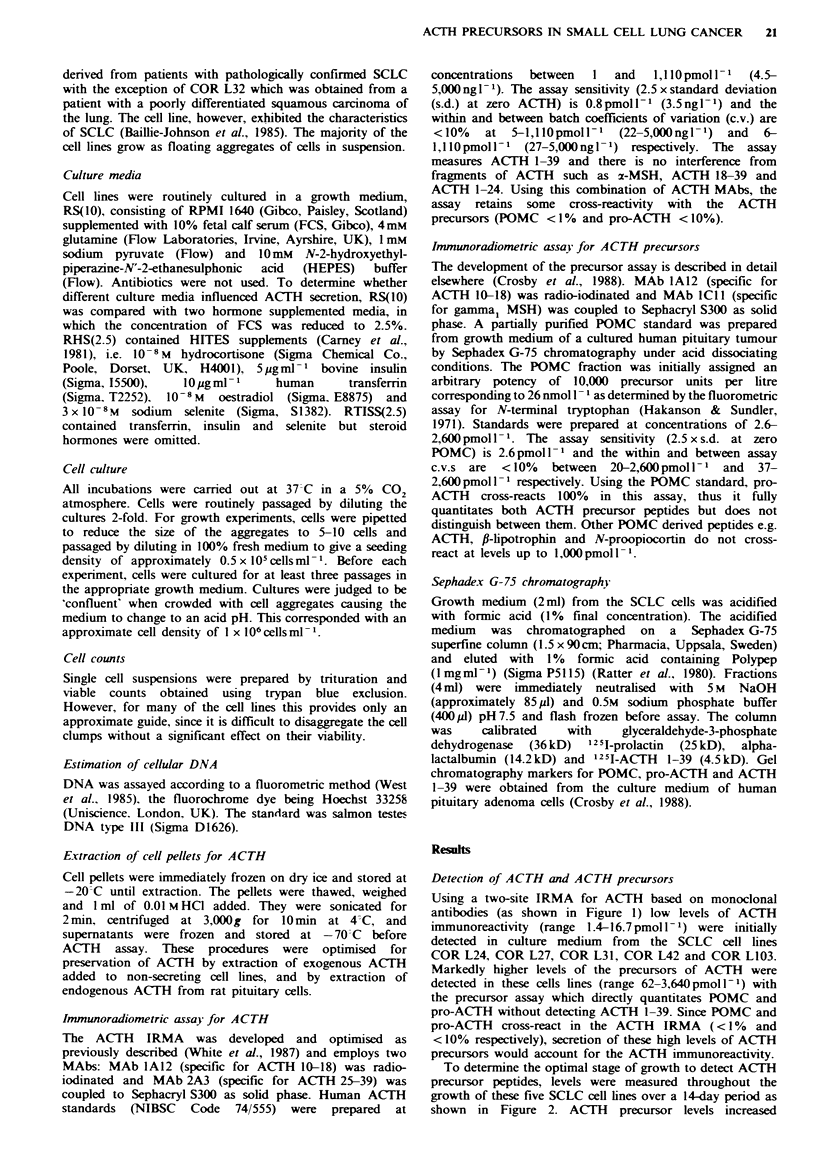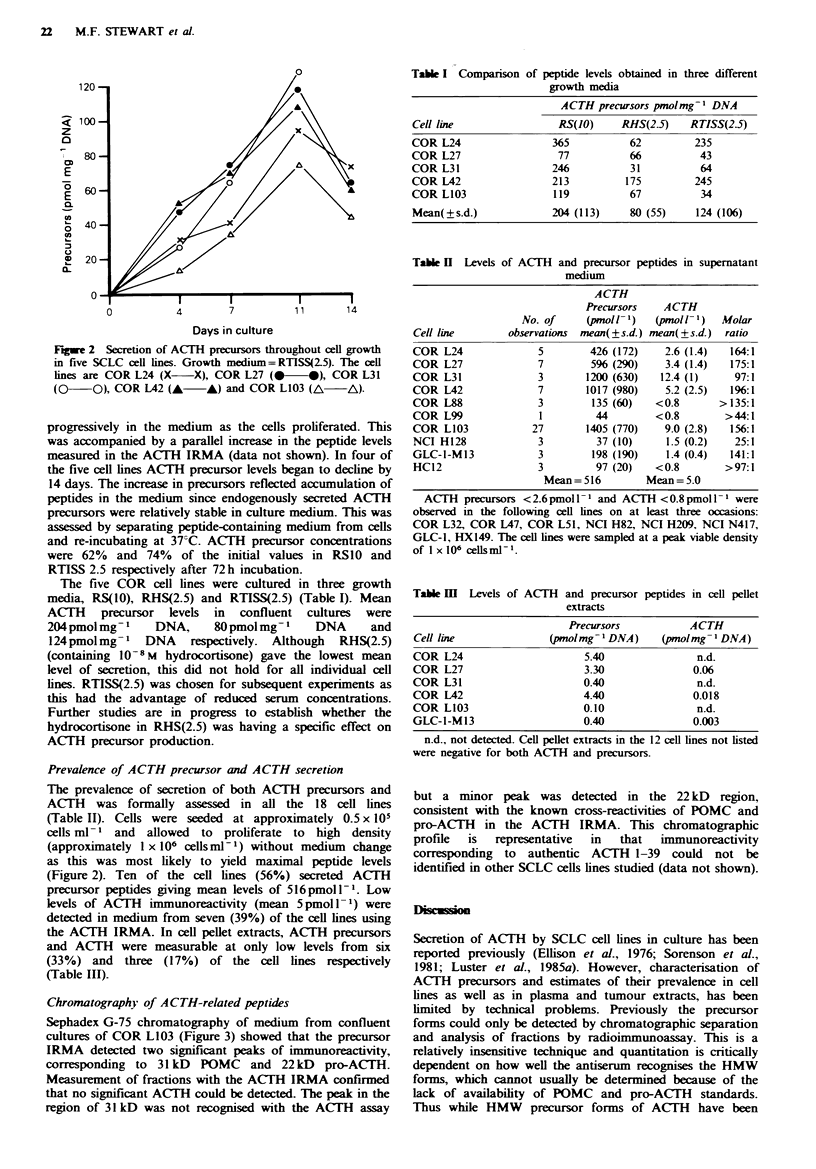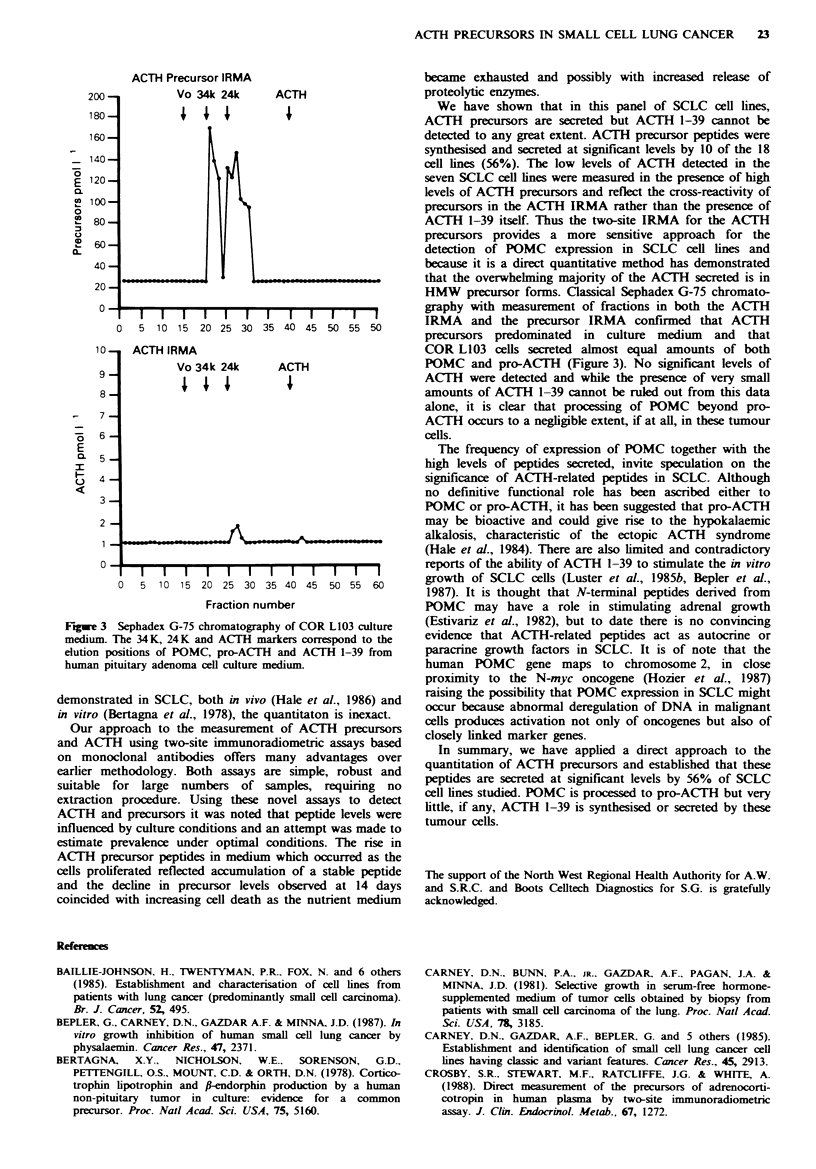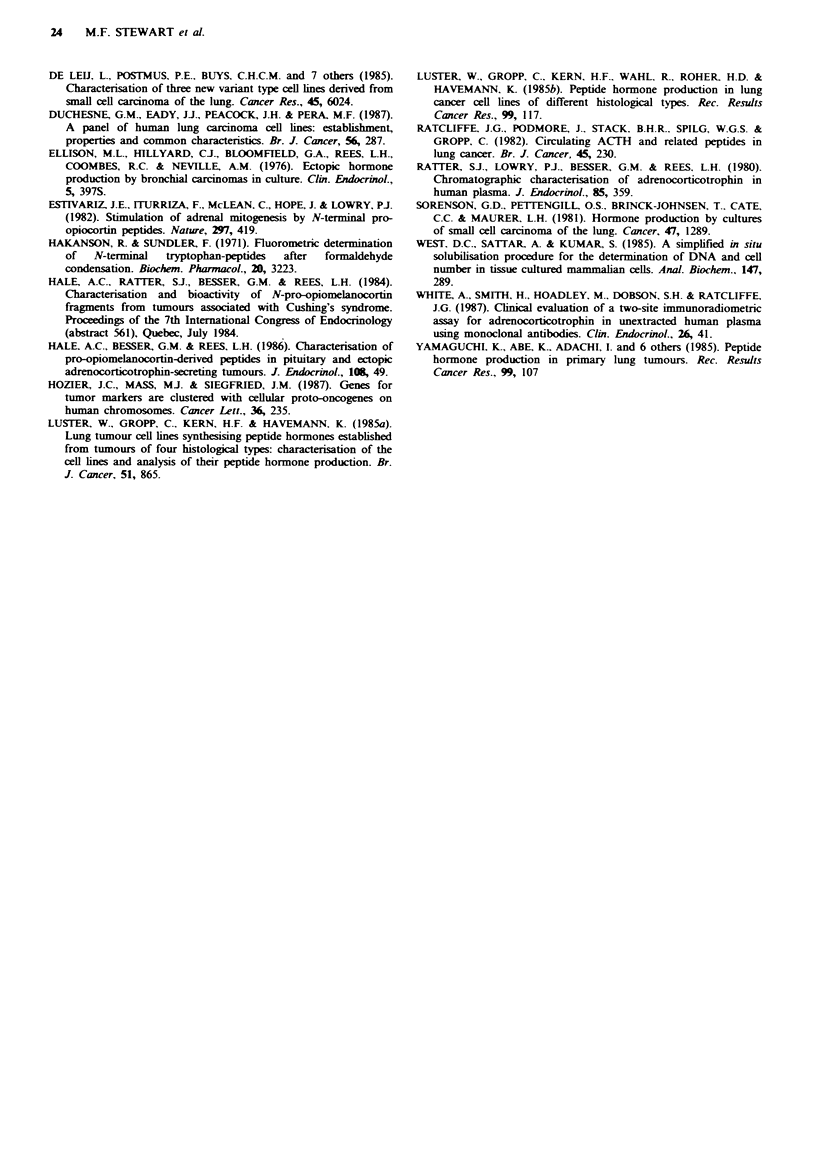# Small cell lung cancer cell lines secrete predominantly ACTH precursor peptides not ACTH.

**DOI:** 10.1038/bjc.1989.212

**Published:** 1989-07

**Authors:** M. F. Stewart, S. R. Crosby, S. Gibson, P. R. Twentyman, A. White

**Affiliations:** University of Manchester, Department of Clinical Biochemistry, Hope Hospital, Salford, UK.

## Abstract

A panel of 18 well characterised human small cell lung cancer (SCLC) cell lines was assessed for the production of adrenocorticotrophin (ACTH) and its precursor peptides, pro-opiomelanocortin (POMC) and pro-ACTH. These precursor peptides were measured directly using a novel two-site immunoradiometric assay (IRMA) based on monoclonal antibodies, in conjunction with a similar IRMA for ACTH 1-39. Significant concentrations of ACTH precursors were secreted by 10 of the 18 cell lines (56%). The low levels of ACTH immunoreactivity detected in seven cell lines could be accounted for by the known cross-reactivity of precursors in the ACTH IRMA. This suggests there is little, if any, processing of ACTH precursors to ACTH. Cell pellet extracts contained undetectable or low levels of ACTH precursors and ACTH, indicating that these peptides are not stored intracellularly. During the growth of the SCLC cells in vitro ACTH precursors accumulated progressively in the culture medium. Thus the combination of a direct assay for the ACTH precursors and the panel of SCLC cell lines provides a valuable in vitro model for the expression of POMC in human tumours.


*Br. J. Cancer* (1989), **60**, 20–24 © The Macmillan Press Ltd., 1989

### Small cell lung cancer cell lines secrete predominantly ACTH precursor peptides not ACTH

M.F. Stewart, S.R. Crosby, S. Gibson, P.R. Twentyman[1] & A. White

*University of Manchester, Department of Clinical Biochemistry, Hope Hospital, Salford M6 8HD, UK; and [1]MRC Clinical Oncology and Radiotherapeutics Unit, Hills Road, Cambridge CB2 2QH, UK.*

**Summary** A panel of 18 well characterised human small cell lung cancer (SCLC) cell lines was assessed for the production of adrenocorticotrophin (ACTH) and its precursor peptides, pro-opiomelanocortin (POMC) and pro-ACTH. These precursor peptides were measured directly using a novel two-site immunoradiometric assay (IRMA) based on monoclonal antibodies, in conjunction with a similar IRMA for ACTH 1-39. Significant concentrations of ACTH precursors were secreted by 10 of the 18 cell lines (56%). The low levels of ACTH immunoreactivity detected in seven cell lines could be accounted for by the known cross-reactivity of precursors in the ACTH IRMA. This suggests there is little, if any, processing of ACTH precursors to ACTH. Cell pellet extracts contained undetectable or low levels of ACTH precursors and ACTH, indicating that these peptides are not stored intracellularly. During the growth of the SCLC cells *in vitro* ACTH precursors accumulated progressively in the culture medium. Thus the combination of a direct assay for the ACTH precursors and the panel of SCLC cell lines provides a valuable *in vitro* model for the expression of POMC in human tumours.

Small cell lung cancer (SCLC) is a hormonally active neoplasm associated with the secretion of a wide range of peptide hormones. Adrenocorticotrophin (ACTH) is a notable example, giving rise to gross metabolic derangement with hypercortisolism and hypokalaemic alkalosis in the ectopic ACTH syndrome. While this is a relatively unusual clinical manifestation of SCLC, occurring in 2–3% of cases, studies investigating the potential role of ACTH as a tumour marker have indicated that 20–30% of patients have elevated plasma levels of immunoreactive (ir)-ACTH, without overt clinical signs of glucocorticoid excess (Ratcliffe *et al.*, 1982). Further, when lung tumours are extracted and assayed for ACTH, some 20% of SCLC tumours yield significant amounts of peptide (Yamaguchi *et al.*, 1985). Thus, the association of ACTH production with SCLC is sufficiently strong to address the question of a functional role in the development or progression of this tumour.

ACTH is synthesised as a high molecular weight (HMW) precursor, pro-opiomelanocortin (POMC, approximate molecular weight 31 kD), which is cleaved to an intermediate peptide, pro-ACTH (molecular weight approximately 22 kD) (Figure 1). It has been suggested that these precursor peptides do not circulate in normal subjects but may circulate in pathological conditions, and particularly in the ectopic ACTH syndrome (Hale *et al.*, 1986). Characterisation of the molecular species has hitherto only been possible by chromatographic separation followed by radioimmunoassay (RIA) of the fractions using antisera to component peptides from the precursor such as ACTH or gamma-melanocyte stimulating hormone (gamma-MSH). We have developed a new approach to quantitating ACTH and the precursor peptides directly in plasma samples. This involved the production of a range of monoclonal antibodies (MAbs) to ACTH and the development of a sensitive two-site immunoradiometric assay (IRMA) for ACTH (White *et al.*, 1987). Subsequently an IRMA for the two precursor peptides, POMC and pro-ACTH has been developed based on MAbs to ACTH and gamma-MSH (Crosby *et al.*, 1988) (Figure 1).

SCLC cell lines are now widely used for the study of tumour biology and provide an appropriate *in vitro* model since *in vivo* studies of hormone secretion are limited by the rapid clinical course of the disease. We have used these two novel assays to establish the prevalence of secretion of ACTH and its precursors in a large panel of cell lines which were established in several different centres. We have examined the influence of culture conditions and the relationship between cell growth and peptide production.

Correspondence: A. White.

**Figure 1** Binding sites of the MAbs used in the IRMAs for ACTH-related peptides. In the precursor IRMA (**a**), the $^{125}$I-MAb-1A12 recognises ACTH 10–18 and the solid phase-linked MAb-1C11 recognises the gamma-MSH sequence. In the ACTH IRMA (**b**), the $^{125}$I-MAb-1A12 recognises ACTH 10–18 and the solid phase-linked MAb-2A3 recognises ACTH 25–39.

### Materials and methods

### *Small cell lung cancer cell lines*

Ten 'COR' cell lines (COR L24, COR L27, COR L31, COR L32, COR L42, COR L47, COR L51, COR L88, COR L99 and COR L103) were established and characterised as previously described (Baillie-Johnson *et al.*, 1985). Four NCI SCLC cell lines (NCI H82, NCI H128, NCI H209 and NCI N417) were made available for study courtesy of Dr F. Cuttitta (Carney *et al.*, 1985). HC12 and HX149 were kindly donated by Dr G. Duchesne, Ludwig Institute for Cancer Research, Sutton, UK (Duchesne *et al.*, 1987). GLC-1 and GLC-1-M13 were a gift from Dr M. Brouwer, Netherlands Cancer Institute, Amsterdam, the Netherlands, and Dr L. de Leij, University of Groningen, the Netherlands (de Leij *et al.*, 1985). All the cell lines were derived from patients with pathologically confirmed SCLC with the exception of COR L32 which was obtained from a patient with a poorly differentiated squamous carcinoma of the lung. The cell line, however, exhibited the characteristics of SCLC (Baillie-Johnson *et al.*, 1985). The majority of the cell lines grow as floating aggregates of cells in suspension.

*Culture media*

Cell lines were routinely cultured in a growth medium, RS(10), consisting of RPMI 1640 (Gibco, Paisley, Scotland) supplemented with 10% fetal calf serum (FCS, Gibco), 4 mM glutamine (Flow Laboratories, Irvine, Ayrshire, UK), 1 mM sodium pyruvate (Flow) and 10 mM *N*-2-hydroxyethyl-piperazine-*N'*-2-ethanesulphonic acid (HEPES) buffer (Flow). Antibiotics were not used. To determine whether different culture media influenced ACTH secretion, RS(10) was compared with two hormone supplemented media, in which the concentration of FCS was reduced to 2.5%. RHS(2.5) contained HITES supplements (Carney *et al.*, 1981), i.e. $10^{-8}$ M hydrocortisone (Sigma Chemical Co., Poole, Dorset, UK, H4001), 5 $\mu$g ml$^{-1}$ bovine insulin (Sigma, I5500), 10 $\mu$g ml$^{-1}$ human transferrin (Sigma, T2252), $10^{-8}$ M oestradiol (Sigma, E8875) and $3 \times 10^{-8}$ M sodium selenite (Sigma, S1382). RTISS(2.5) contained transferrin, insulin and selenite but steroid hormones were omitted.

*Cell culture*

All incubations were carried out at 37°C in a 5% $CO_2$ atmosphere. Cells were routinely passaged by diluting the cultures 2-fold. For growth experiments, cells were pipetted to reduce the size of the aggregates to 5–10 cells and passaged by diluting in 100% fresh medium to give a seeding density of approximately $0.5 \times 10^5$ cells ml$^{-1}$. Before each experiment, cells were cultured for at least three passages in the appropriate growth medium. Cultures were judged to be 'confluent' when crowded with cell aggregates causing the medium to change to an acid pH. This corresponded with an approximate cell density of $1 \times 10^6$ cells ml$^{-1}$.

*Cell counts*

Single cell suspensions were prepared by trituration and viable counts obtained using trypan blue exclusion. However, for many of the cell lines this provides only an approximate guide, since it is difficult to disaggregate the cell clumps without a significant effect on their viability.

*Estimation of cellular DNA*

DNA was assayed according to a fluorometric method (West *et al.*, 1985), the fluorochrome dye being Hoechst 33258 (Uniscience, London, UK). The standard was salmon testes DNA type III (Sigma D1626).

*Extraction of cell pellets for ACTH*

Cell pellets were immediately frozen on dry ice and stored at −20°C until extraction. The pellets were thawed, weighed and 1 ml of 0.01 M HCl added. They were sonicated for 2 min, centrifuged at 3,000 *g* for 10 min at 4°C, and supernatants were frozen and stored at −70°C before ACTH assay. These procedures were optimised for preservation of ACTH by extraction of exogenous ACTH added to non-secreting cell lines, and by extraction of endogenous ACTH from rat pituitary cells.

*Immunoradiometric assay for ACTH*

The ACTH IRMA was developed and optimised as previously described (White *et al.*, 1987) and employs two MAbs: MAb 1A12 (specific for ACTH 10–18) was radio-iodinated and MAb 2A3 (specific for ACTH 25–39) was coupled to Sephacryl S300 as solid phase. Human ACTH standards (NIBSC Code 74/555) were prepared at concentrations between 1 and 1,110 pmol l$^{-1}$ (4.5–5,000 ng l$^{-1}$). The assay sensitivity (2.5 × standard deviation (s.d.) at zero ACTH) is 0.8 pmol l$^{-1}$ (3.5 ng l$^{-1}$) and the within and between batch coefficients of variation (c.v.) are <10% at 5–1,110 pmol l$^{-1}$ (22–5,000 ng l$^{-1}$) and 6–1,110 pmol l$^{-1}$ (27–5,000 ng l$^{-1}$) respectively. The assay measures ACTH 1-39 and there is no interference from fragments of ACTH such as $\alpha$-MSH, ACTH 18–39 and ACTH 1–24. Using this combination of ACTH MAbs, the assay retains some cross-reactivity with the ACTH precursors (POMC <1% and pro-ACTH <10%).

*Immunoradiometric assay for ACTH precursors*

The development of the precursor assay is described in detail elsewhere (Crosby *et al.*, 1988). MAb 1A12 (specific for ACTH 10–18) was radio-iodinated and MAb 1C11 (specific for gamma$_1$ MSH) was coupled to Sephacryl S300 as solid phase. A partially purified POMC standard was prepared from growth medium of a cultured human pituitary tumour by Sephadex G-75 chromatography under acid dissociating conditions. The POMC fraction was initially assigned an arbitrary potency of 10,000 precursor units per litre corresponding to 26 nmol l$^{-1}$ as determined by the fluorometric assay for *N*-terminal tryptophan (Hakanson & Sundler, 1971). Standards were prepared at concentrations of 2.6–2,600 pmol l$^{-1}$. The assay sensitivity (2.5 × s.d. at zero POMC) is 2.6 pmol l$^{-1}$ and the within and between assay c.v.s are <10% between 20–2,600 pmol l$^{-1}$ and 37–2,600 pmol l$^{-1}$ respectively. Using the POMC standard, pro-ACTH cross-reacts 100% in this assay, thus it fully quantitates both ACTH precursor peptides but does not distinguish between them. Other POMC derived peptides e.g. ACTH, $\beta$-lipotrophin and *N*-proopiocortin do not cross-react at levels up to 1,000 pmol l$^{-1}$.

*Sephadex G-75 chromatography*

Growth medium (2 ml) from the SCLC cells was acidified with formic acid (1% final concentration). The acidified medium was chromatographed on a Sephadex G-75 superfine column (1.5 × 90 cm; Pharmacia, Uppsala, Sweden) and eluted with 1% formic acid containing Polypep (1 mg ml$^{-1}$) (Sigma P5115) (Ratter *et al.*, 1980). Fractions (4 ml) were immediately neutralised with 5 M NaOH (approximately 85 $\mu$l) and 0.5M sodium phosphate buffer (400 $\mu$l) pH 7.5 and flash frozen before assay. The column was calibrated with glyceraldehyde-3-phosphate dehydrogenase (36 kD) $^{125}$I-prolactin (25 kD), alpha-lactalbumin (14.2 kD) and $^{125}$I-ACTH 1-39 (4.5 kD). Gel chromatography markers for POMC, pro-ACTH and ACTH 1–39 were obtained from the culture medium of human pituitary adenoma cells (Crosby *et al.*, 1988).

### Results

*Detection of ACTH and ACTH precursors*

Using a two-site IRMA for ACTH based on monoclonal antibodies (as shown in Figure 1) low levels of ACTH immunoreactivity (range 1.4–16.7 pmol l$^{-1}$) were initially detected in culture medium from the SCLC cell lines COR L24, COR L27, COR L31, COR L42 and COR L103. Markedly higher levels of the precursors of ACTH were detected in these cells lines (range 62–3,640 pmol l$^{-1}$) with the precursor assay which directly quantitates POMC and pro-ACTH without detecting ACTH 1–39. Since POMC and pro-ACTH cross-react in the ACTH IRMA (<1% and <10% respectively), secretion of these high levels of ACTH precursors would account for the ACTH immunoreactivity.

To determine the optimal stage of growth to detect ACTH precursor peptides, levels were measured throughout the growth of these five SCLC cell lines over a 14-day period as shown in Figure 2. ACTH precursor levels increased

**Figure 2** Secretion of ACTH precursors throughout cell growth in five SCLC cell lines. Growth medium = RTISS(2.5). The cell lines are COR L24 (X——X), COR L27 (●——●), COR L31 (○——○), COR L42 (▲——▲) and COR L103 (△——△).

progressively in the medium as the cells proliferated. This was accompanied by a parallel increase in the peptide levels measured in the ACTH IRMA (data not shown). In four of the five cell lines ACTH precursor levels began to decline by 14 days. The increase in precursors reflected accumulation of peptides in the medium since endogenously secreted ACTH precursors were relatively stable in culture medium. This was assessed by separating peptide-containing medium from cells and re-incubating at 37°C. ACTH precursor concentrations were 62% and 74% of the initial values in RS10 and RTISS 2.5 respectively after 72 h incubation.

The five COR cell lines were cultured in three growth media, RS(10), RHS(2.5) and RTISS(2.5) (Table I). Mean ACTH precursor levels in confluent cultures were 204 pmol mg$^{-1}$ DNA, 80 pmol mg$^{-1}$ DNA and 124 pmol mg$^{-1}$ DNA respectively. Although RHS(2.5) (containing $10^{-8}$ M hydrocortisone) gave the lowest mean level of secretion, this did not hold for all individual cell lines. RTISS(2.5) was chosen for subsequent experiments as this had the advantage of reduced serum concentrations. Further studies are in progress to establish whether the hydrocortisone in RHS(2.5) was having a specific effect on ACTH precursor production.

### *Prevalence of ACTH precursor and ACTH secretion*

The prevalence of secretion of both ACTH precursors and ACTH was formally assessed in all the 18 cell lines (Table II). Cells were seeded at approximately $0.5 \times 10^5$ cells ml$^{-1}$ and allowed to proliferate to high density (approximately $1 \times 10^6$ cells ml$^{-1}$) without medium change as this was most likely to yield maximal peptide levels (Figure 2). Ten of the cell lines (56%) secreted ACTH precursor peptides giving mean levels of 516 pmol l$^{-1}$. Low levels of ACTH immunoreactivity (mean 5 pmol l$^{-1}$) were detected in medium from seven (39%) of the cell lines using the ACTH IRMA. In cell pellet extracts, ACTH precursors and ACTH were measurable at only low levels from six (33%) and three (17%) of the cell lines respectively (Table III).

### *Chromatography of ACTH-related peptides*

Sephadex G-75 chromatography of medium from confluent cultures of COR L103 (Figure 3) showed that the precursor IRMA detected two significant peaks of immunoreactivity, corresponding to 31 kD POMC and 22 kD pro-ACTH. Measurement of fractions with the ACTH IRMA confirmed that no significant ACTH could be detected. The peak in the region of 31 kD was not recognised with the ACTH assay

**Table I** Comparison of peptide levels obtained in three different growth media

| | *ACTH precursors pmol mg$^{-1}$ DNA* | | |
|---|---|---|---|
| *Cell line* | *RS(10)* | *RHS(2.5)* | *RTISS(2.5)* |
| COR L24 | 365 | 62 | 235 |
| COR L27 | 77 | 66 | 43 |
| COR L31 | 246 | 31 | 64 |
| COR L42 | 213 | 175 | 245 |
| COR L103 | 119 | 67 | 34 |
| Mean(±s.d.) | 204 (113) | 80 (55) | 124 (106) |

**Table II** Levels of ACTH and precursor peptides in supernatant medium

| *Cell line* | *No. of observations* | *ACTH Precursors (pmol l$^{-1}$) mean(±s.d.)* | *ACTH (pmol l$^{-1}$) mean(±s.d.)* | *Molar ratio* |
|---|---|---|---|---|
| COR L24 | 5 | 426 (172) | 2.6 (1.4) | 164:1 |
| COR L27 | 7 | 596 (290) | 3.4 (1.4) | 175:1 |
| COR L31 | 3 | 1200 (630) | 12.4 (1) | 97:1 |
| COR L42 | 7 | 1017 (980) | 5.2 (2.5) | 196:1 |
| COR L88 | 3 | 135 (60) | <0.8 | >135:1 |
| COR L99 | 1 | 44 | <0.8 | >44:1 |
| COR L103 | 27 | 1405 (770) | 9.0 (2.8) | 156:1 |
| NCI H128 | 3 | 37 (10) | 1.5 (0.2) | 25:1 |
| GLC-1-M13 | 3 | 198 (190) | 1.4 (0.4) | 141:1 |
| HC12 | 3 | 97 (20) | <0.8 | >97:1 |
| | | Mean = 516 | Mean = 5.0 | |

ACTH precursors <2.6 pmol l$^{-1}$ and ACTH <0.8 pmol l$^{-1}$ were observed in the following cell lines on at least three occasions: COR L32, COR L47, COR L51, NCI H82, NCI H209, NCI N417, GLC-1, HX149. The cell lines were sampled at a peak viable density of $1 \times 10^6$ cells ml$^{-1}$.

**Table III** Levels of ACTH and precursor peptides in cell pellet extracts

| *Cell line* | *Precursors (pmol mg$^{-1}$ DNA)* | *ACTH (pmol mg$^{-1}$ DNA)* |
|---|---|---|
| COR L24 | 5.40 | n.d. |
| COR L27 | 3.30 | 0.06 |
| COR L31 | 0.40 | n.d. |
| COR L42 | 4.40 | 0.018 |
| COR L103 | 0.10 | n.d. |
| GLC-1-M13 | 0.40 | 0.003 |

n.d., not detected. Cell pellet extracts in the 12 cell lines not listed were negative for both ACTH and precursors.

but a minor peak was detected in the 22 kD region, consistent with the known cross-reactivities of POMC and pro-ACTH in the ACTH IRMA. This chromatographic profile is representative in that immunoreactivity corresponding to authentic ACTH 1–39 could not be identified in other SCLC cells lines studied (data not shown).

### Discussion

Secretion of ACTH by SCLC cell lines in culture has been reported previously (Ellison *et al.*, 1976; Sorenson *et al.*, 1981; Luster *et al.*, 1985*a*). However, characterisation of ACTH precursors and estimates of their prevalence in cell lines as well as in plasma and tumour extracts, has been limited by technical problems. Previously the precursor forms could only be detected by chromatographic separation and analysis of fractions by radioimmunoassay. This is a relatively insensitive technique and quantitation is critically dependent on how well the antiserum recognises the HMW forms, which cannot usually be determined because of the lack of availability of POMC and pro-ACTH standards. Thus while HMW precursor forms of ACTH have been

**Figure 3** Sephadex G-75 chromatography of COR L103 culture medium. The 34 K, 24 K and ACTH markers correspond to the elution positions of POMC, pro-ACTH and ACTH 1–39 from human pituitary adenoma cell culture medium.

demonstrated in SCLC, both *in vivo* (Hale *et al.*, 1986) and *in vitro* (Bertagna *et al.*, 1978), the quantitaton is inexact.

Our approach to the measurement of ACTH precursors and ACTH using two-site immunoradiometric assays based on monoclonal antibodies offers many advantages over earlier methodology. Both assays are simple, robust and suitable for large numbers of samples, requiring no extraction procedure. Using these novel assays to detect ACTH and precursors it was noted that peptide levels were influenced by culture conditions and an attempt was made to estimate prevalence under optimal conditions. The rise in ACTH precursor peptides in medium which occurred as the cells proliferated reflected accumulation of a stable peptide and the decline in precursor levels observed at 14 days coincided with increasing cell death as the nutrient medium became exhausted and possibly with increased release of proteolytic enzymes.

We have shown that in this panel of SCLC cell lines, ACTH precursors are secreted but ACTH 1–39 cannot be detected to any great extent. ACTH precursor peptides were synthesised and secreted at significant levels by 10 of the 18 cell lines (56%). The low levels of ACTH detected in the seven SCLC cell lines were measured in the presence of high levels of ACTH precursors and reflect the cross-reactivity of precursors in the ACTH IRMA rather than the presence of ACTH 1–39 itself. Thus the two-site IRMA for the ACTH precursors provides a more sensitive approach for the detection of POMC expression in SCLC cell lines and because it is a direct quantitative method has demonstrated that the overwhelming majority of the ACTH secreted is in HMW precursor forms. Classical Sephadex G-75 chromatography with measurement of fractions in both the ACTH IRMA and the precursor IRMA confirmed that ACTH precursors predominated in culture medium and that COR L103 cells secreted almost equal amounts of both POMC and pro-ACTH (Figure 3). No significant levels of ACTH were detected and while the presence of very small amounts of ACTH 1–39 cannot be ruled out from this data alone, it is clear that processing of POMC beyond pro-ACTH occurs to a negligible extent, if at all, in these tumour cells.

The frequency of expression of POMC together with the high levels of peptides secreted, invite speculation on the significance of ACTH-related peptides in SCLC. Although no definitive functional role has been ascribed either to POMC or pro-ACTH, it has been suggested that pro-ACTH may be bioactive and could give rise to the hypokalaemic alkalosis, characteristic of the ectopic ACTH syndrome (Hale *et al.*, 1984). There are also limited and contradictory reports of the ability of ACTH 1–39 to stimulate the *in vitro* growth of SCLC cells (Luster *et al.*, 1985*b*, Bepler *et al.*, 1987). It is thought that *N*-terminal peptides derived from POMC may have a role in stimulating adrenal growth (Estivariz *et al.*, 1982), but to date there is no convincing evidence that ACTH-related peptides act as autocrine or paracrine growth factors in SCLC. It is of note that the human POMC gene maps to chromosome 2, in close proximity to the N-*myc* oncogene (Hozier *et al.*, 1987) raising the possibility that POMC expression in SCLC might occur because abnormal deregulation of DNA in malignant cells produces activation not only of oncogenes but also of closely linked marker genes.

In summary, we have applied a direct approach to the quantitation of ACTH precursors and established that these peptides are secreted at significant levels by 56% of SCLC cell lines studied. POMC is processed to pro-ACTH but very little, if any, ACTH 1–39 is synthesised or secreted by these tumour cells.

The support of the North West Regional Health Authority for A.W. and S.R.C. and Boots Celltech Diagnostics for S.G. is gratefully acknowledged.

### References

BAILLIE-JOHNSON, H., TWENTYMAN, P.R., FOX, N. and 6 others (1985). Establishment and characterisation of cell lines from patients with lung cancer (predominantly small cell carcinoma). *Br. J. Cancer*, **52**, 495.

BEPLER, G., CARNEY, D.N., GAZDAR A.F. & MINNA, J.D. (1987). *In vitro* growth inhibition of human small cell lung cancer by physalaemin. *Cancer Res.*, **47**, 2371.

BERTAGNA, X.Y., NICHOLSON, W.E., SORENSON, G.D., PETTENGILL, O.S., MOUNT, C.D. & ORTH, D.N. (1978). Corticotrophin lipotrophin and β-endorphin production by a human non-pituitary tumor in culture: evidence for a common precursor. *Proc. Natl Acad. Sci. USA*, **75**, 5160.

CARNEY, D.N., BUNN, P.A., JR., GAZDAR, A.F., PAGAN, J.A. & MINNA, J.D. (1981). Selective growth in serum-free hormone-supplemented medium of tumor cells obtained by biopsy from patients with small cell carcinoma of the lung. *Proc. Natl Acad. Sci. USA*, **78**, 3185.

CARNEY, D.N., GAZDAR, A.F., BEPLER, G. and 5 others (1985). Establishment and identification of small cell lung cancer cell lines having classic and variant features. *Cancer Res.*, **45**, 2913.

CROSBY, S.R., STEWART, M.F., RATCLIFFE, J.G. & WHITE, A. (1988). Direct measurement of the precursors of adrenocorticotropin in human plasma by two-site immunoradiometric assay. *J. Clin. Endocrinol. Metab.*, **67**, 1272.

DE LEIJ, L., POSTMUS, P.E., BUYS, C.H.C.M. and 7 others (1985). Characterisation of three new variant type cell lines derived from small cell carcinoma of the lung. *Cancer Res.*, **45**, 6024.

DUCHESNE, G.M., EADY, J.J., PEACOCK, J.H. & PERA, M.F. (1987). A panel of human lung carcinoma cell lines: establishment, properties and common characteristics. *Br. J. Cancer*, **56**, 287.

ELLISON, M.L., HILLYARD, C.J., BLOOMFIELD, G.A., REES, L.H., COOMBES, R.C. & NEVILLE, A.M. (1976). Ectopic hormone production by bronchial carcinomas in culture. *Clin. Endocrinol.*, **5**, 397S.

ESTIVARIZ, J.E., ITURRIZA, F., McLEAN, C., HOPE, J. & LOWRY, P.J. (1982). Stimulation of adrenal mitogenesis by *N*-terminal pro-opiocortin peptides. *Nature*, **297**, 419.

HAKANSON, R. & SUNDLER, F. (1971). Fluorometric determination of *N*-terminal tryptophan-peptides after formaldehyde condensation. *Biochem. Pharmacol.*, **20**, 3223.

HALE, A.C., RATTER, S.J., BESSER, G.M. & REES, L.H. (1984). Characterisation and bioactivity of *N*-pro-opiomelanocortin fragments from tumours associated with Cushing's syndrome. Proceedings of the 7th International Congress of Endocrinology (abstract 561), Quebec, July 1984.

HALE, A.C., BESSER, G.M. & REES, L.H. (1986). Characterisation of pro-opiomelanocortin-derived peptides in pituitary and ectopic adrenocorticotrophin-secreting tumours. *J. Endocrinol.*, **108**, 49.

HOZIER, J.C., MASS, M.J. & SIEGFRIED, J.M. (1987). Genes for tumor markers are clustered with cellular proto-oncogenes on human chromosomes. *Cancer Lett.*, **36**, 235.

LUSTER, W., GROPP, C., KERN, H.F. & HAVEMANN, K. (1985*a*). Lung tumour cell lines synthesising peptide hormones established from tumours of four histological types: characterisation of the cell lines and analysis of their peptide hormone production. *Br. J. Cancer*, **51**, 865.

LUSTER, W., GROPP, C., KERN, H.F., WAHL, R., ROHER, H.D. & HAVEMANN, K. (1985*b*). Peptide hormone production in lung cancer cell lines of different histological types. *Rec. Results Cancer Res.*, **99**, 117.

RATCLIFFE, J.G., PODMORE, J., STACK, B.H.R., SPILG, W.G.S. & GROPP, C. (1982). Circulating ACTH and related peptides in lung cancer. *Br. J. Cancer*, **45**, 230.

RATTER, S.J., LOWRY, P.J., BESSER, G.M. & REES, L.H. (1980). Chromatographic characterisation of adrenocorticotrophin in human plasma. *J. Endocrinol.*, **85**, 359.

SORENSON, G.D., PETTENGILL, O.S., BRINCK-JOHNSEN, T., CATE, C.C. & MAURER, L.H. (1981). Hormone production by cultures of small cell carcinoma of the lung. *Cancer*, **47**, 1289.

WEST, D.C., SATTAR, A. & KUMAR, S. (1985). A simplified *in situ* solubilisation procedure for the determination of DNA and cell number in tissue cultured mammalian cells. *Anal. Biochem.*, **147**, 289.

WHITE, A., SMITH, H., HOADLEY, M., DOBSON, S.H. & RATCLIFFE, J.G. (1987). Clinical evaluation of a two-site immunoradiometric assay for adrenocorticotrophin in unextracted human plasma using monoclonal antibodies. *Clin. Endocrinol.*, **26**, 41.

YAMAGUCHI, K., ABE, K., ADACHI, I. and 6 others (1985). Peptide hormone production in primary lung tumours. *Rec. Results Cancer Res.*, **99**, 107